# Optimizing hormonal and amino acid combinations for enhanced cell proliferation and cell cycle progression in bovine mammary epithelial cells

**DOI:** 10.5713/ab.23.0199

**Published:** 2023-08-28

**Authors:** Hyuk Cheol Kwon, Hyun Su Jung, Do Hyun Kim, Jong Hyeon Han, Seo Gu Han, Dong Hyun Keum, Seong Joon Hong, Sung Gu Han

**Affiliations:** 1 Department of Food Science and Biotechnology of Animal Resources, Konkuk University, Seoul 05029, Korea

**Keywords:** Bovine Mammary Epithelial Cell, Cell Cycle, Cell Proliferation, Essential Amino Acid, Hormone, Optimization

## Abstract

**Objective:**

The number of bovine mammary epithelial cells (BMECs) is closely associated with the quantity of milk production in dairy cows; however, the optimal levels and the combined effects of hormones and essential amino acids (EAAs) on cell proliferation are not completely understood. Thus, the purpose of this study was to determine the optimal combination of individual hormones and EAAs for cell proliferation and related signaling pathways in BMECs.

**Methods:**

Immortalized BMECs (MAC-T) were treated with six hormones (insulin, cortisol, progesterone, estrone, 17β-estradiol, and epidermal growth factor) and ten EAAs (arginine, histidine, leucine, isoleucine, threonine, tryptophan, lysine, methionine, phenylalanine, and valine) for 24 h.

**Results:**

Cells were cultured in a medium containing 10% fetal bovine serum (FBS) as FBS supplemented at a concentration of 10% to 50% showed a comparable increase in cell proliferation rate. The optimized combination of four hormones (insulin, cortisol, progesterone, and 17β-estradiol) and 20% of a mixture of ten EAAs led to the highest cell proliferation rate, which led to a significant increase in cell cycle progression at the S and G2/M phases, in the protein levels of proliferating cell nuclear antigen and cyclin B1, cell nucleus staining, and in cell numbers.

**Conclusion:**

The optimal combination of hormones and EAAs increased BMEC proliferation by enhancing cell cycle progression in the S and G/2M phases. Our findings indicate that optimizing hormone and amino acid levels has the potential to enhance milk production, both in cell culture settings by promoting increased cell numbers, and in dairy cows by regulating feed intake.

## INTRODUCTION

The mammary gland comprises luminal and basal epithelia. The basal epithelium consists mainly of myoepithelial cells and a smaller population of stem cells. In contrast, the luminal epithelium comprises epithelial cells that play a crucial role in milk production [1]. Milk productivity and secretory activity are closely related to the number of mammary epithelial cells (MECs) and the ratio of cell proliferation to cell loss [2]. Therefore, various strategies have been investigated to increase cell proliferation and decrease cell loss in order to enhance milk yield.

Bovine milk quality and productivity in dairy cows depend on interactions between various internal and external factors [3,4]. Internal factors include body development, body conformation, constitution, genetic factors, breed, and lactation stage. External factors include season, diet, geographic origin, health conditions, and severe physical conditions such as heat stress and water deprivation [3,5]. Consequently, numerous studies have been conducted with the aim of increasing the milk yield via the interaction and regulation of inherent and extrinsic factors [5,6]. In particular, inherent factors have been reported to be more closely related to milk production in dairy cows than extrinsic factors are.

The growth of the mammary glands for milk production occurs mainly during body development, including prepuberty, postpuberty, gestation, and lactation [7]. Each growth and development stage is hormonally regulated by endocrine, autocrine, paracrine, and intracellular factors [8]. Thus, hormone regulation is a major internal factor in milk yield enhancement. Hormones, such as insulin (I), cortisol (C), progesterone (P), estrone (E1), 17β-estradiol (E2), and epidermal growth factor (EGF), induce cell growth and proliferation in mammary cells [8–11]; however, previous studies have focused mainly on increasing cell proliferation through the addition of hormones to the mammary glands.

The intake and balance of appropriate nutrients are other major internal factors that play crucial roles in the growth and development of mammary glands [12]. Specifically, the proper provision of amino acids (AAs) is closely associated with milk synthesis and the structural development of mammary glands [13]. Supplementation with essential amino acids (EAAs) has been shown to increase cell viability in bovine and murine MECs, thereby resulting in growth and development of the mammary gland [14–16]. Nevertheless, previous studies have evaluated cell viability through single or multiple additions of EAAs to cells.

Although previous studies have shown that some hormones and EAAs increase the cell viability of MECs in mammary glands, the combined and interactive effects of various hormones and EAAs on cell proliferation of bovine MECs (BMECs) have not been comprehensively studied. In particular, there is a lack of data on how an optimized combination of hormones and EAAs increases cell proliferation and regulates the underlying cellular mechanisms. Therefore, this study aimed to investigate the effects of individual hormones, EAAs, and their optimal combinations on cell proliferation and the underlying cellular mechanisms in BMECs. Our data may serve as a crucial foundation for cultivating primary BMECs and the subsequently synthesis of milk components within a cell culture framework.

## MATERIALS AND METHODS

### Chemical and reagents

Hormones (I, C, P, E1, E2, and EGF) were purchased from Sigma-Aldrich (St. Louis, MO, USA). L-Arginine (Arg), L-histidine (His), L-leucine (Leu), L-isoleucine (Ile), L-threonine (Thr), L-tryptophan (Trp), L-lysine (Lys), L-methionine (Met), L-phenylalanine (Phe), and L-valine (Val) were purchased from Sigma-Aldrich (USA). Dulbecco’s modified Eagle medium/nutrient mixture F12 (DMEM/F12) was obtained from Gibco (Grand Island, NY, USA). Fetal bovine serum (FBS), penicillin/streptomycin (P/S), and trypsin-ethylenediaminetetraacetic acid (EDTA) solutions were purchased from WELGENE Inc. (Gyeongsan, Daegu, Korea). Phosphate-buffered saline (PBS) was obtained from Lonza (Walkersville, MD, USA).

### Cell culture and treatment

Immortalized BMECs (MAC-T) were cultured using DMEM/F12 medium supplemented with 10% FBS and 1% P/S in a humidified 5% carbon dioxide incubator at 37°C. The cells were maintained in a T-75 cell culture flask and supplied with fresh medium every two days. MAC-T cells were sub-cultured in T-75 cell culture flasks and seeded into 6-well, 12-well, and 96-well cell culture plates. The cells were exposed to various concentrations of FBS (0% to 100%) for 24 h. In addition, the cells were treated with six hormones and ten EAAs for 24 h. The concentration ranges were as follows: hormones (I, 0 to 10,000 ng/mL; C, 0 to 5,000 ng/mL; P, 0 to 5,000 ng/mL; E1, 0 to 5,000 ng/mL; E2, 0 to 1,000 ng/mL; and EGF, 0 to 1,000 ng/mL) and EAAs (Arg, 0 to 7.0 mM; His, 0 to 1.0 mM; Leu, 0 to 1.2 mM; Ile, 0 to 1.2 mM; Thr, 0 to 1.2 mM; Trp, 0 to 1.5 mM; Lys, 0 to 2.0 mM; Met, 0 to 1.5 mM; Phe, 0 to 1.5 mM; and Val, 0 to 1.5 mM).

### Cell proliferation assay

Cell proliferation was evaluated using 3-(4,5-dimethylthiazole-2yl)-2,5-diphenyl-2H-tetrazolium bromide (MTT; Amresco, Solon, OH, USA). MAC-T cells were seeded at a density of 5×10^3^ cells/well in 96-well plates and treated with each hormone and EAAs at various concentrations for 24 h. Afterwards, the cells were exchanged with 110 μL of a fresh medium containing 10 μL of MTT solution (5 mg/mL in PBS) and then incubated at 37°C for 3 h. Next, 90 μL of the medium was discarded, then 180 μL of acidic isopropanol was added to solubilize the insoluble formazan crystals. Subsequently, the 96-well cell culture plates were placed in incubator at 37°C for 1 h. Afterward, the optical density (OD) was measured at 570 nm and 630 nm using an Epoch spectrophotometer (BioTek Instruments, Winooski, VT, USA). To obtain a corrected OD value, the OD at 630 nm was calculated from the OD value at 570 nm. Cell proliferation was expressed as a fold-change using the following formula:


Cell proliferation (fold)=(OD experimental sample/OD control sample)

Cell numbers were evaluated using trypan blue solution (Gibco, Carlsbad, CA, USA). The cells were cultured at a density of 0.2×10^6^ cells/well in 12-well cell culture plates and exposed to combinations of hormones and EAAs for 24 h. The cells were dissociated using trypsin-EDTA solution and centrifuged at 600×g for 10 min. Cell pellets were stained with trypan blue solution, and the number of viable cells was determined using a hemocytometer (Hausser Scientific, Horsham, PA, USA).

### Cell cycle analysis

The cells were starved with serum-free media for 24 h and treated with the optimal combination of hormones (I, 1 ng/mL; C, 5 ng/mL; P, 10 ng/mL; and E2, 0.1 ng/mL) and the optimal combination of hormones and 20% level of EAAs (Arg, 5.0 mM; His, 1.0 mM; Leu, 0.9 mM; Ile, 0.6 mM; Thr, 0.9 mM; Trp, 1.2 mM; Lys, 2.0 mM; Met, 1.2 mM; Phe, 1.5 mM; and Val, 1.5 mM) for 24 h. After treatment, the cells were detached using trypsin-EDTA solution and collected in 1.7-mL microtubes. The cells were fixed in 70% ethanol at 4°C for 16 h. Next, the cells were suspended in PBS of 500 μL supplemented with RNase A (2 mg/mL, final concertation; Sigma-Aldrich, USA) and incubated at 37°C for 1 h. Subsequently, propidium iodide (0.1 mg/mL, final concentration; Sigma-Aldrich, USA) was added to the cells in 1.7-mL microtubes for staining, and the stained cells were analyzed for cell cycle distribution using a CytoFlex Flow Cytometry Analyzer (Beckman Coulter, Brea, CA, USA).

### Protein extraction and western blot

The protein expression of proliferating cell nuclear antigen (PCNA) and cyclin B1 was evaluated using western blotting. Protein samples were lysed using a radioimmunoprecipitation assay buffer (Elpis Biotech, Daejeon, Korea) supplemented with a protease inhibitor cocktail (Abbkine Inc., Wuhan, China). The cell extracts were centrifuged at 17,000×g at 4°C for 20 min. Proteins were quantified using a bicinchoninic acid protein assay kit (Thermo Fisher Scientific, Waltham, MA, USA). Each protein sample was loaded into a sample well with 5% stacking and 10% separating acrylamide gels using a Mini-PROTEAN Tetra Vertical Electrophoresis Cell (Bio-Rad Laboratories, Hercules, CA, USA). The proteins were then transferred onto nitrocellulose membranes (Bio-Rad Laboratories, USA) using a Semi-Dry Electrophoretic Transfer Cell (GE Healthcare Biosciences, Chicago, IL, USA). Thereafter, the membranes were blocked using 5% of nonfat milk buffer diluted in Tris-buffered saline with Tween 20 buffer (TBST) at 20°C for 90 min and incubated with anti-PCNA (Santa Cruz Biotechnology, Dallas, TX, USA), anti-cyclin B1 (Cell Signaling Technology, Danvers, MA, USA), and anti-glyceraldehyde 3-phosphate dehydrogenase (GAPDH; Merck Millipore, Darmstadt, Germany) primary antibody at 4°C for three days. The membranes were then washed three times with TBST and incubated with goat anti-rabbit IgG conjugated with horseradish peroxidase (Enzo Life Sciences, Lausen, Switzerland) at 20°C for 90 min. Protein expression signals were visualized using an enhanced chemiluminescence detection reagent (Thermo Fisher Scientific, USA). The intensity of the bands was quantified using the ImageJ software (National Institutes of Health, Bethesda, MD, USA).

### Nuclear staining assay

Cell nuclei were stained with 4′,6-diamidino-2-phenylindole (DAPI; Sigma-Aldrich, USA) to observe cell proliferation. The cells were exposed to the optimal combination of hormones and 20% EAAs for 24 h and fixed with 4% paraformaldehyde at 20°C for 15 min. After fixation, the cells were permeabilized using PBS-0.1% Triton X-100 and then stained with DAPI (1 μg/mL in distilled water) for 10 min. The nuclei (blue fluorescence) were imaged and captured using Nikon Eclipse Ti2-U and Nikon Eclipse Ts2R cameras (Nikon Co., Ltd., Tokyo, Japan). Fluorescence intensities were quantified using the ImageJ software.

### Statistical analysis

Experimental data are presented as mean±standard error of the mean. One-way analysis of variance with Duncan’s new multiple range test was performed to analyze the statistical significance using SPSS-PASW statistics software (version 22.0 SPSS Inc., Chicago, IL, USA). Statistical significance is defined when p values are less than 0.05.

## RESULTS

### Effect of FBS on cell proliferation in MAC-T cells

**Figure 1:**
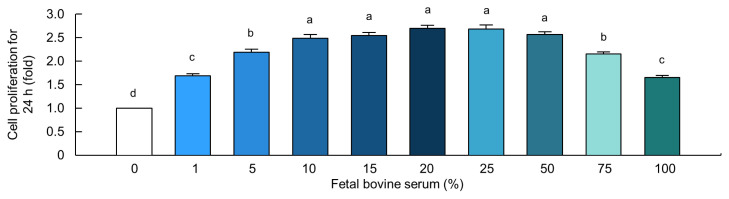
Fetal bovine serum (FBS) induced the increase of cell proliferation in MAC-T cells. Cell proliferation rate in cells treated with FBS at 0%, 1%, 5%, 10%, 15%, 20%, 25%, 50%, 75%, and 100% for 24 h (*n* = 5). The data are expressed as mean±standard error of the mean. ^a–d^ Different superscript letters present significant differences (p<0.05).

Cells treated with 0%, 1%, 5%, 10%, 15%, and 20% FBS for 24 h showed a progressive increase in proliferation. However, when cells were exposed to higher FBS concentrations (25%, 50%, 75%, and 100%) for the same duration, cell proliferation gradually declined (Figure 1). Importantly, FBS at concentrations between 10% and 50% showed significantly higher cell proliferation rates than the control did (p<0.05). FBS at a concentration of 10% was used for further experiments to minimize its cost.

### Effect of individual hormone and optimal hormone combination on cell proliferation in MAC-T cells

**Figure 2:**
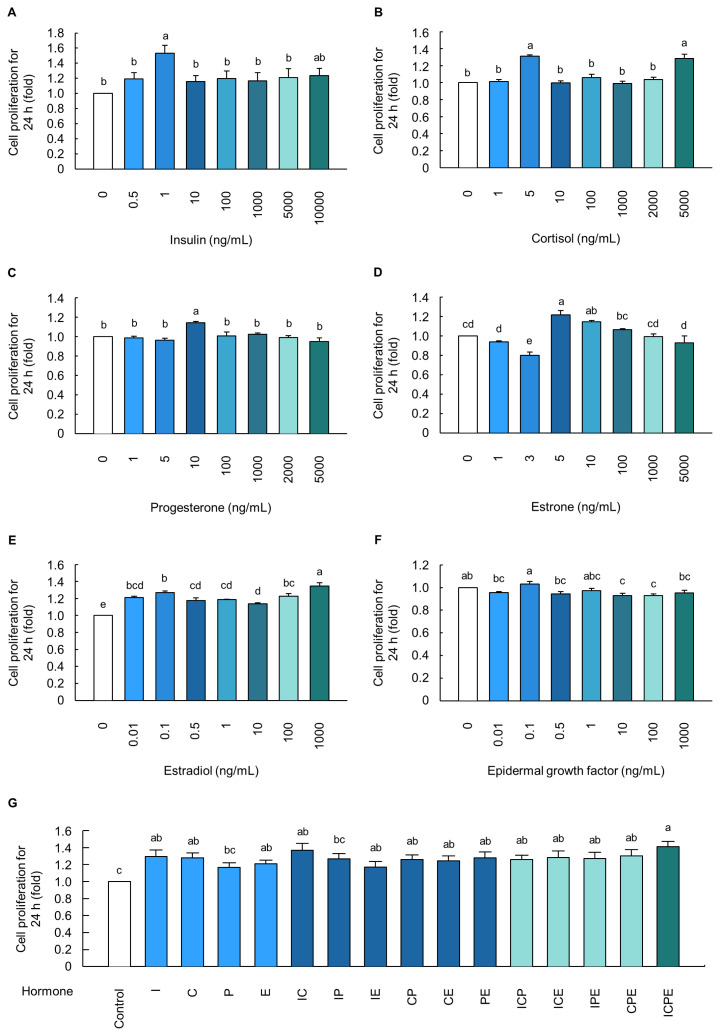
Individual hormones and optimized combination of hormones induced the increase of cell proliferation in MAC-T cells. Cell proliferation rate in cells treated with (A) insulin (0 to 10,000 ng/mL), (B) cortisol (0 to 5,000 ng/mL), (C) progesterone (0 to 5,000 ng/mL), (D) estrone (0 to 5,000 ng/mL), (E) estradiol (0 to 1,000 ng/mL), and (F) epidermal growth factor (0 to 1,000 ng/mL) for 24 h (*n* = 5). (G) The cell proliferation rate was measured in cells treated to the various combination of four hormones including insulin (I; 1 ng/mL), cortisol (C; 5 ng/mL), progesterone (P; 10 ng/mL), and estradiol (E; 0.1 ng/mL) for 24 h (*n* = 10). The data are expressed as mean±standard error of the mean. ^a–e^ Different superscript letters present significant differences (p<0.05).

Cell proliferation was significantly increased in cells treated with I (1 ng/mL), C (5 and 5,000 ng/mL), P (10 ng/mL), E1 (5 and 10 ng/mL), and E2 (0.1 and 1,000 ng/mL) for 24 h, compared to that in control cells (p<0.05; Figure 2A–2E). Notably, the C, E1, and E2 hormones showed a similar cell proliferation rate even at lower concentrations (5, 5, and 0.1 ng/mL, respectively), compared to that in the cells treated with high concentrations (5,000, 10, and 1,000 ng/mL, respectively) (Figure 2B, 2D, and 2E, respectively). Furthermore, because E1 is a precursor that is known to be converted to E2 in cells, E1 was excluded from the optimal hormone combination. In addition, there was no significant difference in EGF-induced cell proliferation (p>0.05; Figure 2F). Thus, the four hormones I (1 ng/mL), C (5 ng/mL), P (10 ng/mL), and E (0.1 ng/mL) were selected to optimize the combination of hormones, and the cells treated with the combination of the four hormones had a 1.413-fold higher cell proliferation rate than the control cells (p<0.05; Figure 2G).

### Effect of individual EAAs and the optimal EAA combination on cell proliferation in MAC-T cells

**Figure 3:**
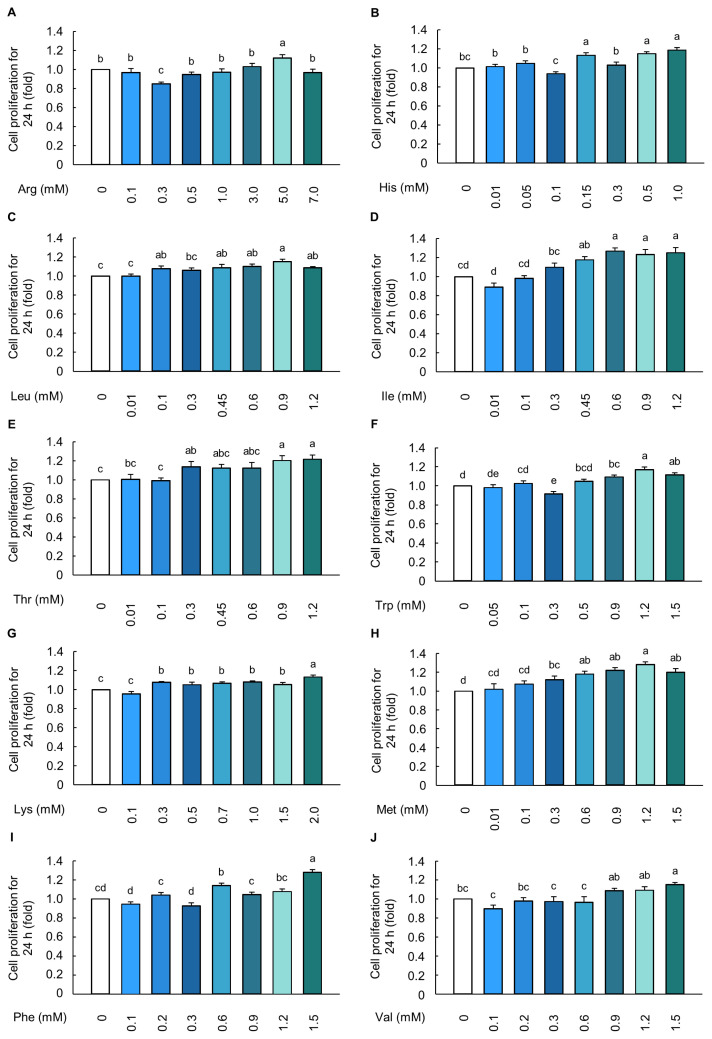
Individual essential amino acids induced the increase in cell proliferation of MAC-T cells. Cell proliferation rate in cells treated with (A) arginine (Arg, 0 to 7.0 mM), (B) histidine (His, 0 to 1.0 mM), (C) leucine (Leu, 0 to 1.2 mM), (D) isoleucine (Ile, 0 to 1.2 mM), (E) threonine (Thr, 0 to 1.2 mM), (F) tryptophan (Trp, 0 to 1.5 mM), (G) lysine (Lys, 0 to 2.0 mM), (H) methionine (Met, 0 to 1.5 mM), (I) phenylalanine (Phe, 0 to 1.5 mM), and (J) valine (Val, 0 to 1.5 mM) for 24 h (*n* = 5). The data are expressed as mean±standard error of the mean. ^a–e^ Different superscript letters present significant differences (p<0.05).

**Figure 4:**
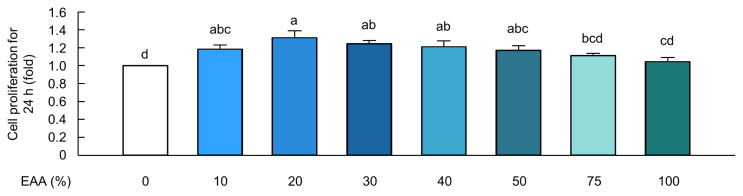
Optimal combination of hormones and essential amino acids induced the increase of cell proliferation in MAC-T cells. Cell proliferation rate in MAC-T cells treated with the selected optimal level of hormones and varying essential amino acid (EAA) levels, ranging from 0 to 100% of the selected individual EAAs such as arginine (5.0 mM), histidine (1.0 mM), leucine (0.9 mM), isoleucine (0.6 mM), threonine (0.9 mM), tryptophan (1.2 mM), lysine (2 mM), methionine (1.2 mM), phenylalanine (1.5 mM), and valine (1.5 mM) for 24 h (*n* = 5). The data are expressed as mean± standard error of the mean. ^a–d^ Different superscript letters present significant differences (p<0.05).

MAC-T cells were exposed to ten individual EAAs along with a combination of four hormones for 24 h. Cells were treated with Arg (5.0 mM), His (1.0 mM), Leu (0.9 mM), Ile (0.6 mM), Thr (0.9 mM), Trp (1.2 mM), Lys (2.0 mM), Met (1.2 mM), Phe (1.5 mM), and Val (1.5 mM) showed significantly higher cell proliferation rates than the control cells (p<0.05; Figure 3). The cells were then treated with a mixture of ten EAAs at varying EAA levels (ranging from 0% to 100% of the selected individual EAA concentrations), along with the hormones (Figure 4). Cells treated with the optimal combination of hormones and the EAAs at 20% of the selected EAA concentrations showed significantly higher cell proliferation rates than control cells (p<0.05; Figure 4).

### Effect of optimal hormones and EAAs on the cell cycle and cell cycle-related protein levels in MAC-T cells

**Figure 5:**
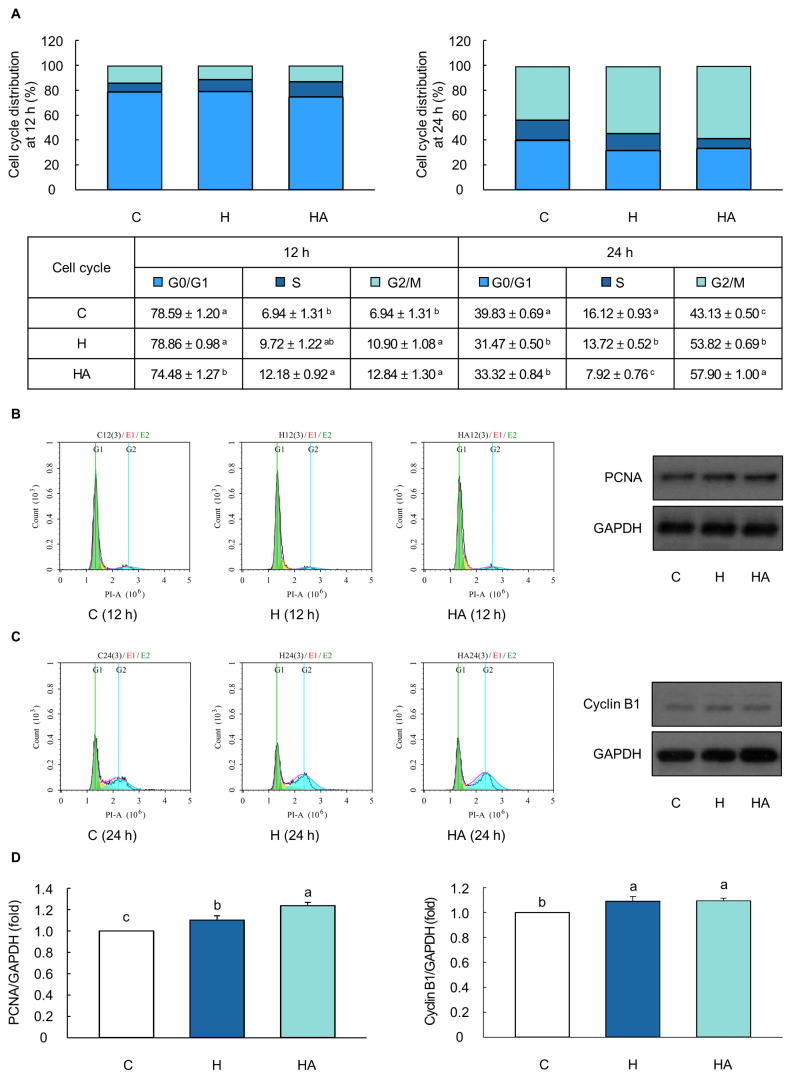
Optimal combination of hormones and essential amino acids induced the increase of cell cycle progression in MAC-T cells. (A) Cell cycle distribution ratio in cell treated with the optimal hormones level (H) and hormones and 20% level of essential amino acids (HA) for 12 and 24 h, respectively (*n* = 6). (B–D) Cell cycle distribution images (*n* = 6) and protein levels of proliferating cell nuclear antigen (PCNA) and cyclin B1 (*n* = 3) in cells treated with H and HA for 12 and 24 h, respectively. Glyceraldehyde-3-phosphate dehydrogenase (GAPDH) was used as a housekeeping protein control. Representative images are selected from six or three independent replicates. The data are expressed as mean±standard error of the mean. ^a–c^ Different superscript letters present significant differences (p<0.05).

Treatment of cells with the optimal hormones (H) and hormones with 20% EAAs (HA) significantly increased the S and G2/M phases during the cell cycle distribution (p<0.05; Figure 5A). The protein expression level of PCNA significantly increased in cells treated with H and HA for 12 h, compared to that in control cells (p<0.05; Figure 5B and 5D). Furthermore, after 24 h of exposure to the optimal concentrations of H and HA, the cells exhibited a significantly higher protein expression level of cyclin B1 than the control cells did (p<0.05; Figure 5C and 5D).

### Effect of optimal hormones and EAAs on cell proliferation in MAC-T cells

**Figure 6:**
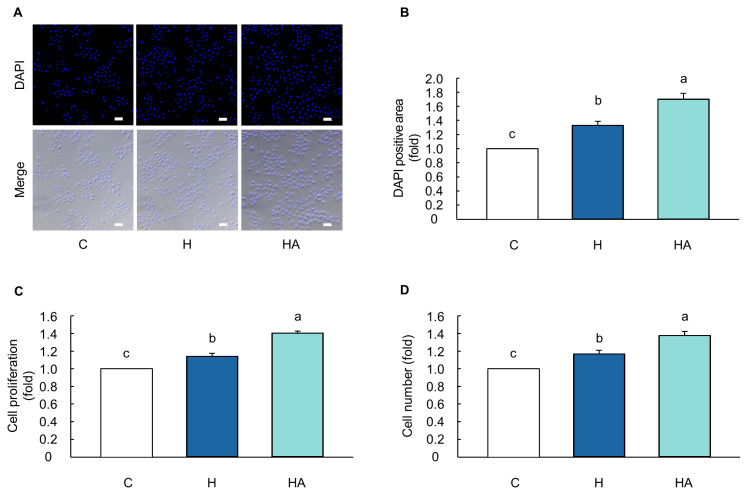
Optimal combination of hormones and essential amino acids induced the increase of DAPI-stained cell nuclei and cell numbers in MAC-T cells. (A) Fluorescence images and (B) blue fluorescence positive area ratio of DAPI stained nucleus in cells treated with the optimal hormones (H) and hormones and 20% level of essential amino acids (HA) for 24 h (*n* = 3). (C) Cell proliferation rate and (D) cell number in cells treated with H and HA for 24 h (*n* = 3–4). The magnification of the images was 100×. Scale bar indicates 100 μM. Representative images are selected from three independent replicates. The data are expressed as mean±standard error of the mean. ^a–c^ Different superscript letters present significant differences (p<0.05).

Cells exposed to the optimal H and HA concentrations for 24 h showed significantly larger DAPI-stained areas than the control cells (p<0.05; Figure 6A and 6B). Moreover, the cell proliferation in the optimal H- and HA-treated cells for 24 h significantly increased by up to 1.142- and 1.406-fold, respectively, compared to that in the control cells (p<0.05; Figure 6C). Furthermore, the optimized H- and HA-treated cells for 24 h showed 1.170- and 1.379-fold higher cell numbers, respectively, than the control cells (p<0.05; Figure 6D).

## DISCUSSION

Because the quantity of milk produced from bovine mammary glands is closely associated with the number of MECs [17], an increase in cell proliferation of BMECs has been reported to directly affect milk productivity [18]. Various factors related to the development of bovine mammary glands have been used to increase cell proliferation and milk production [19,20]. A previous study showed that some hormones, such as growth hormone, prolactin, I, C, P, and E2, increase cell proliferation and milk production in lactating cow [21]. Moreover, treatment of mammalian cells with various AAs as carbon and nitrogen sources increased cell proliferation [22]. Previous studies demonstrated that hormones and amino acids increase cell proliferation. However, the effects of specific combinations of hormones and AAs on BMEC proliferation have not been thoroughly investigated. Therefore, we aimed to comprehensively evaluate the effects of hormones, AAs, and their combination, particularly on cell proliferation and associated cellular mechanisms, using the BMEC line MAC-T.

The circulatory system plays a crucial role in providing hormones and nutrients for the development and growth of mammary glands [23]. Therefore, the concentrations of hormones (I, C, P, E1, E2, and EGF) used in this study were chosen based on bovine blood hormone concentrations (Figure 2). The experimental design involved the use of approximate blood hormone concentrations as the starting point, followed by the inclusion of higher concentrations. In detail, the concentration of I was 0.35 ng/mL at 15 months of age and 0.416 to 0.625 ng/mL from pregnancy to lactation in Holstein–Friesian dairy cows [24,25]. The concentration of C was 9 ng/mL at calving and was maintained at 3 to 5 ng/mL in crossbred Karan Fries and Holstein–Friesian dairy cows [26,27]. The concentration of P in Holstein–Friesian dairy cows during the postpartum period increased from approximately 0.5 ng/mL on day 0 to 3.5 ng/mL on day 50 [28]. Moreover, the concentrations of E1 and E2 increased to 3.5 ng/mL and 0.55 ng/mL, respectively, before parturition and remained constant at 0.05 ng/mL and 0.025 ng/mL, respectively, after parturition in bovine blood [29]. EGF was determined based on a concentration of 10 ng/mL, which was used for the differentiation of lactating MECs because there have been no previous studies on the change in plasma EGF during bovine parturition [30]. Herein, cells treated with I, C, P, E1, E2, and EGF displayed higher levels of cell proliferation than control cells at concentrations of 1, 5, 10, 5, 0.1, and 0.1 ng/mL, respectively. Interestingly, the individual hormone concentrations that increased cell proliferation were largely consistent with hormone concentrations in bovine blood. However, our data showed that EGF had no influence on cell proliferation, and because E2 is transformed from E1 in cells [31,32], hormones I, C, P, and E2 were selected as the final combination for the evaluation of cell proliferation. Collectively, our data demonstrate that the combination of I, C, P, and E2 is optimal for promoting BMEC proliferation.

AAs are crucial regulatory factors in metabolism and homeostasis. They have been found to have a direct effect on the growth and development of the mammary glands and milk synthesis [33]. Previous studies have shown that EAAs increase BMEC proliferation via the mammalian target of rapamycin (mTOR) signaling pathway, thereby leading to the enhancement of milk protein [14,34]. Our results also showed that the treatment of cells with ten individual EAAs increased the BMEC proliferation at a concentration of 5.0 mM of Arg, 1.0 mM of His, 0.9 mM Leu, 0.6 mM of Ile, 0.9 mM of Thr, 1.2 mM of Trp, 2 mM of Lys, 1.2 mM of Met, 1.5 mM of Phe, and 1.5 mM of Val, compared to control cells (Figure 3). The concentration ranges and optimal proliferation levels of ten individual EAAs were determined based on previous studies that investigated increases in cell numbers and cell proliferation-related signaling markers in BMECs. For instance, a previous study reported that treating BMECs with 3.2 mM Arg increased the expression of casein milk protein via the mTOR signaling pathway, which is related to cell proliferation [35]. Treating BMECs with 1.2 mM His for 24 h resulted in a significant increase in cell proliferation [34]. MAC-T cells treated with 0.90 mM Leu and 0.63 mM Ile for 24 h showed increased cell proliferation [36]. Treatment with 1 or 2 mM Lys and 1.44 mM Met also increased BMEC proliferation and casein synthesis via the mTOR pathway [34,37]. In addition, Phe and Val treatment of MAC-T cells at various concentrations (0 to 1.5 mM) enhanced protein synthesis and milk protein-related mTOR signaling pathways [38]. Taken together, these data indicate that the ten individual EAAs used herein had a concentration range similar to those used in previous studies. Notably, our study showed that the combination of hormones and 20% of ten EAAs resulted in the highest cell proliferation rate in MAC-T cells (Figure 4). These data are consistent with previous studies in which excessive EAAs decreased cell proliferation and milk protein expression in BMECs [34–37]. Furthermore, excessive EAA causes the production of harmful metabolites, a decrease in protein synthesis, and an increase in protein degradation, which reduces cell proliferation by downregulating the mTOR pathway [16,39]. Consequently, our data indicated that the combination of the four hormones and 20% EAAs was optimal for achieving the highest cell proliferation rate in MAC-T cells.

Cell proliferation is controlled by cell cycle progression; thus, an increase in cell proliferation is closely associated with changes in the proportion of cell cycle phases, such as G1, S, and G2/M [40]. DNA replication during the S phase occurs via the interaction between PCNA and p21 [41]. After completion of the S phase, the repair of DNA double-strand breaks, synthesis of proteins and RNA, segregation of chromosomes, and cell division, an increase in cyclin-dependent kinase 1/cyclin B1 complexes occurs in the G2/M phase [42]. Therefore, increased levels of PCNA and cyclin B1 indicate a significant increase in cell proliferation. Herein, the optimal combination of H and HA increased the S and G2/M phases of the cell cycle and the protein expression levels of PCNA and cyclin B1 in BMECs after 12 and 24 h, respectively (Figure 5). Consistent with this study, the peptide hormone (kisspeptin-10) and optimal EAAs had resulted in higher cell viability with an increase in S and G2/M phases compared to that of control cells in BMECs [14,43]. In addition, previous studies have reported that the increase in the S and G2/M phases during the progression of the cell cycle promotes cell proliferation along with an increase in PCNA and cyclin B1 levels in BMECs and mouse mesenchymal stem cells [44–46]. Moreover, our data showed that H- and HA-treated cells had higher DAPI-stained areas, thereby indicating an increase in cell proliferation and cell numbers compared to that in control cells (Figure 6). Collectively, our data showed that an optimal combination of hormones and EAAs increased cell proliferation and the number of MAC-T cells through cell cycle progression of the S and G2/M phases.

## CONCLUSION

This study demonstrated that five individual hormones and ten individual EAAs increased MAC-T cell proliferation at the following concentrations (1 ng/mL I, 5 ng/mL C, 10 ng/mL P, 5 ng/mL E1, 0.1 ng/mL E2, 5.0 mM Arg, 1.0 mM His, 0.9 mM Leu, 0.6 mM Ile, 0.9 mM Thr, 1.2 mM Trp, 2.0 mM Lys, 1.2 mM Met, 1.5 mM Phe, and 1.5 mM Val). The optimal combination of four hormones (I, C, P, and E2) and a level of 20% of the ten EAA mixture resulted in 1.41-fold higher cell proliferation, 1.38-fold higher cell number, and 1.7-fold higher DAPI-positive area than the control. This combination increased cell proliferation through cell cycle progression of the S and G/2 phases and increased protein expression of PCNA and cyclin B1. Although further experiments using more elaborate statistical tools, such as response surface methodology, may be required to evaluate multiple components and their interactions, this study could serve as the initial data to optimize hormone and AA combinations in MAC-T cell proliferation. Our findings offer valuable insights into hormones and nutrition for enhancing both BMEC proliferation and milk production under cell culture conditions and in dairy cows. Furthermore, our data could serve as a crucial foundation for the synthesis of milk components within a cell culture framework.
